# Phenolic Compounds Analysis of Root, Stalk, and Leaves of Nettle

**DOI:** 10.1100/2012/564367

**Published:** 2012-04-19

**Authors:** Semih Otles, Buket Yalcin

**Affiliations:** Food Engineering Department, Engineering Faculty, Ege University, 35100 Bornova/Izmir, Turkey

## Abstract

Types of nettles (*Urtica dioica*) were collected from different regions to analyze phenolic compounds in this research. Nettles are specially grown in the coastal part. According to this kind of properties, nettle samples were collected from coastal part of (Mediterranean, Aegean, Black sea, and Marmara) Turkey. Phenolic profile, total phenol compounds, and antioxidant activities of nettle samples were analyzed. Nettles were separated to the part of root, stalk, and leaves. Then, these parts of nettle were analyzed to understand the difference of phenolic compounds and amount of them. Nettle (root, stalk and leaves) samples were analyzed by using High-Performance Liquid Chromatography with Diode-Array Detection (HPLC-DAD) to qualitative and quantitative determination of the phenolic compounds. Total phenolic components were measured by using Folin-Ciocalteu method. The antioxidant activity was measured by using DPPH (2,2-diphenyl-1-picrylhydrazyl) which is generally used for herbal samples and based on single electron transfer (SET).

## 1. Introduction

Nettle (*Urtica dioica L.*), naturally found in pathway, field, and wildwood. Nettles are grown in mild climate areas, bottom of barriers, ruins and grassy places, between cultivated plants, street, and water runnels. This plant prefers nutrient riches and lighted places, hot and mild climate. It has broad distribution of the world. It is also grown in the different region, of Turkey. Some local names of nettle are “dalagan, dizlagan, agdalak, and isirgi”. Nettle is annual or perennial, herbaceous plant. This plant which is specially grown in Black Sea region has reach chemical composition. It is used as drug, food, fibrous, dye, and cosmetic from centuries. Numbers of medical and pharmacologic researches about nettle are increased day by day. On the other hand, nettle has valuable fibrous content which is light, elegant, long, and resistant. In Turkey, nettle could be an alternative product in Black Sea region in which it could be grown easily [1].

Nettle has dark green leaves, root, stem, serration, and stinging nettle. The nettle flowers are small and green. It could give flowers from May to September. The fruits of nettle are arid and single germ. It has two species. Both of them have 2–4 cm long, oval, and core shape leaves. Fresh nettle could cause blushing and burning of skin when it is touched [2].

Plants could be used as a cure for different types of diseases for centuries. In recent years, usage of plant is increased. In Turkey, plants could be used as household and herbal remedy. Phenolic compounds could be defined as biologically active and herbal and have positive effects on health. The scientific researches are increased about the positive effect of phenolic compounds into coronary heart disease and high blood pressure, diabetes, cancer, inflammative, viral and parasitic disease, psychotic disorders [3].

**Systematic properties of nettle could be classified as [4];**

Scientific name: *Urtica dioica. *
Traditional name: Nettle.
Genus: Urticaceae.
Used parts: root, stalk, and leaves.
Usage as a food: tea (root, stalk, and leaves), vegetable dish (stalk and leaves), salad (stalk and leaves), and so forth.

A research about nettle indicates that it has wide field of usage as household remedy in Italy from gastrointestinal diseases to rheumatism pains [5]. Another research indicates household remedy usage in Moorish, stalk, and leaves of nettle used in treatment of diabetes, hypertension, astringent, antirheumatic, diuretic, antidiuretic, and cholagogue [6].

Nettle has agglutinin, acetophenone, alkaloids, acetylcholine, chlorogenic acid, butyric acid, chlorophylll, caffeic acid, carbonic acid, choline, histamine, coumaric acid, formic acid, pantothenic acid, kaempferol, coproporphyrin, lectin, lecithin, lignan, linoleic and linolenic acids, palmitic acid, xanthophyll, quercetin, quinic acid, serotonin, stigmasterol, terpenes, violaxanthin, and succinic acid in its chemical content. Nettle also contains 2,5% fatty substance, 14–17% albumins, and 18% protein in dry matter. Seeds of nettle contain 8–10% fixed oil. 1 kg fresh plant contains 130 mg vitamin C, 730 mg carotene, and oxalate. Stinging hair of nettle contains formic acid, histamine, and acetylcholine. Leaves of nettle contain provitamin A, vitamin B_1_, K, xanthophylls, and sistosterin and ashes of nettle contain 6,3% ferric oxide, potassium, calcium, and silicium [1].

Analysis of methanolic extracts of nettle was made by using Gas Chromatography-Mass Spectrometry (GC-MS) method; vanillic acid, homovanillic acid, 2-hydroxycinnamic acid, 4-hydroxycinnamic acid, and ferulic acid were found [7]. Methanolic extract of nettle leaves analysis was made by using Reverse Phase High-Performance Liquid Chromatography (RP-HPLC) method, UV detector; syringic acid, gallic acid, and ferulic acid were found [8].

In a research about nettle root, analysis was done by using extract which were prepared in different pH of organic solvent and by using GC-MS method. The results indicate that roots have 18 different phenolic components and 8 different lignan components. But the chemical composition of these components was not identified in this study [9]. In another research, some kind of components (ferulic acid 20 *μ*g/g, homovanillly alcohol 8 *μ*g/g, and p-coumaric acid 5 *μ*g/g) were identified by using isocratically fractionate, commercial *Urtica dioica* root extracts without hydrolisation [10]. In another research, 7 flavonoid glycosides (kaempferol-3-O-glycoside, quercetin-3-O-glycoside, isorhamnetin-3-O glycoside, quercetin-3-O-rutinoside, isorhamnetin-3-O-rutinoside, kaempferol-3-O-rutinoside and isorhamnetin-3-O-neohesperidoside) were isolated from *Urtica dioica* flowers. Structure of these components was identified by using chromatographic and spectroscopic method [11].

Methanolic extracts of nettle leaves and stalks were studied about anthocyanin glycosides, and 3 different components (pelargonidin xylobioside, pelargonidin monoxyloside, and another component which give pelargonidin, D-glucose, and L-rhamnose after acid hydrolysis) were isolated [12]. In addition to the 7 different components, quercetin and rutin flavonoids were also found in leaves and stalks of nettle by using modern spectroscopic (Nuclear magnetic resonance-NMR-, Mass Spectrometry-MS-, etc.) methods [13].

By using Supercritical Fluids Extraction (SFE) method in which liquid CO_2_, different pressure, and temperature profiles were used, chlorophylll a, chlorophylll b, *β*-carotene, and lutein components were found in nettle leaves [14]. In another research chlorophylll a and chlorophyll b were isolated from nettles, which could be used as a coloring agents (E140) in the field of drug and food. The most efficient process was identified as 4-step extraction and nettle was firstly dried at 40°C, stored at dark place in plastic bags at 4°C [15].

Linoleic acid, palmitic acid, oleic acid, palmitoleic acid, stearic acid, gadoleic acid, and erucic acid were found in nettle roots analysis by using GC about fatty acids composition [16]. In a research (−)-epi catechin and (+)-catechin were found in nettle leaves by using RP-HPLC method and UV detector. Nettle has some antimicrobial effects as its phenolic contents. Nettle could inhibit *Staphylococcus aureus*, *Bacillus cereus,* and *Listeria monocytogenes* [8].

A plant mix “Antidiabetis” is used which could decrease blood glucose level and contains root and other parts of nettle in Croatia [17]. Nettle is also used as an antioxidant which could decrease muscular contraction as analgesic. It also shows antimicrobial effect and it has some benefit on gastric mucosal damage [18]. In household remedy nettle is used against muscle paralysis. According to this point, nettles effect on proteolytic activity of *Botulinum* neurotoxins was studied. Nettle leaves extract could inhibit *Botulinum* neurotoxin serotype A light chain protease activity without competition, but it has no effect on serotype B [19].

The aim of this study was to analyze phenolic component and antioxidant activity of nettle, which is grown in coastal part of Turkey (Mediterranean, Aegean, Black sea, and Marmara). In this paper the nettle was taken apart (root, stalk, and leaves), and these parts were analyzed separately. These analyses were done to indicate differences of nettles between regions and parts. The samples were coded as Mediterranean (01-Adana, 07-Antalya, 07F-Fethiye, 32-Isparta), Aegean (09Y-wild sample from Aydın, 09-Aydın, 20-Denizli, 35-Izmir, 45A-Alasehir, 45S-Salihli, 45T-Turgutlu, 48-Mugla), Black Sea (52-Ordu, 53-Rize, 55-Samsun, 61-Trabzon, 74-Bartın) and Marmara (16-Bursa, 41-Kocaeli) which are name of cities. On the other hand, the moisture content of nettles was also analyzed. Firstly nettles were extracted. Then, analysis was done by using extract of nettles. Phenolic profiles of nettle were determined by using HPLC (High-Performance Liquid Chromatography) method.

## 2. Materials and Methods

### 2.1. Materials

#### 2.1.1. Nettle Samples

Samples were collected from Aegean, Black Sea, Marmara, and Mediterranean region. These samples were rapidly washed and dried. Then, they were separated into root, stalk and leaves. After this process, part of samples was kept in a zip lock bag at −20°C for inhibition of air contact before analysis.

#### 2.1.2. Sample Preparation and Extraction

Required amount of the samples were taken and then whittle into small particles to increase extraction yield.

After trials of different types of extraction method [20, 21], 80% methanol-water mixture was used as an extraction liquid. 1 g samples were taken from each of samples. 10 mL extraction liquid was added into the samples and extracted during 1 h at 50°C. The extraction was done in closed vessels to inhibit loss of the extraction liquid. After extraction process, the eluate was filtered.

#### 2.1.3. Chemical Materials

*Standards*-Gallic acid (Sigma, G7384), ferulic acid (Fluka, 42280), rutin (Sigma, R5143), myricetin (Sigma, M6760), syringic acid (Sigma, S6881), caffeic acid (Sigma, C0625), chlorogenic acid (Sigma, C3878), quercetin hydrate (Sigma, 337951), p-coumaric acid (Sigma, C9008), kaempferol (Sigma, K0133), catechin hydrate (Fluka, 22110), fumaric acid (Fluka, 47910), vanillic acid (Fluka, 94770), naringin (Sigma, N1376), ellagic acid (Sigma, E2250), and isorhamnetin (Fluka, 17794) were used.

*Chemicals*-Folin-ciocalteau phenol reactive (Sigma-Aldrich, E9252), acetic acid (Panreac, 361008), sodium carbonate (J.T. Baker, 2024), DPPH (2,2 diphenil, 1, picrylhydrazyl) (Sigma, D9132), and *Chromatographically pure gradient solvent*-Methanol (Labscan, A17C11) were used.

### 2.2. Method

#### 2.2.1. Moisture Analysis

Moisture analysis of samples was done by using “Fruit and Vegetables Moisture Analysis” method [22].

According to method we have the following:

- 3 gr of nettle from each part (root, stalk, and leaves) was weighed (0,001 of accuracy).
- Firstly weighing bottles were kept in drying oven for 1 h at 103 ± 2°C and then samples were put in weighing bottles and kept in drying oven for 4 h at 103 ± 2°C.
- Then samples were taken out from drying oven and then kept in desiccators for 30 min to reach the room temperature.
- After being weighed, samples were put again in drying oven for 1 h to dry.
- These drying periods (1 h) were done until reaching the constant weighing (7 h).
- The moisture contents of samples were measured by using the following formula:
  (1) $$\text{Moisture}\%=\left[\frac{m_{1}-m_{2}}{m_{1}}\right]*100,$$
  where *m*_1_ is initial weigh of sample, *m*_2_ is final weigh of sample.

#### 2.2.2. Total Phenolic Content Analysis

Total phenolic content analysis of nettle extracts was done by using Folin-Ciocalteau (FC) method [23, 24]. The results were expressed as gallic acid equivalents in milligrams per gram of dry matter. The solutions was as follows:

- FC Reactive,
- 7% Na_2_CO_3_ solution,
- gallic acid standard solution (50–100–150–200–300 ppm) preparing by using 80% methanol.

According to the method, we have the following.

- 250 *μ*L FC reactive was added into 50 *μ*L nettle extract or standard solution.
- This mixture was stayed at room temperature in dark place for 5 min.
- At the end of this period, 750 *μ*L 7% Na_2_CO_3_ solution was added. By this way, phenolics hydroxyl groups could give H to water.
- This mixture was completed to 5 mL with pure water.
- Then, mixture was stayed at room temperature in dark place for 120 min for reaction.
- Samples and standards absorbance was measured at 760 nm.
- For blank solution instead of 50 *μ*L extract, 80% methanol solution was added.
- Total phenolic content was measured with calibration curve by using gallic acid equivalent standards.

#### 2.2.3. DPPH Antioxidant Capacity Analysis Method

Nettle extracts antioxidant capacity analysis was done by using DPPH radical degradation activity method [25].

According to the method, we have the following.

- 6 × 10^-5 ^M (molar) DPPH radical was prepared daily by using pure methanol.
- 2 *μ*L methanolic DPPH solution was added into 100 *μ*L sample extract or standard solution.
- This mixture was stayed in dark place for 20 min.
- At the end of this time, absorbance was measured at 515 nm.
- Pure methanol was used as blank solution.
- For control solution, instead of 100 *μ*L extract 100 *μ*L pure water was used.
- Sample extract antioxidant capacities were measured by using calibration curve which was prepared by using different concentrations (10–100 ppm) of gallic acid solution.

#### 2.2.4. HPLC Analysis of Phenolic Component

**Figure 1:**
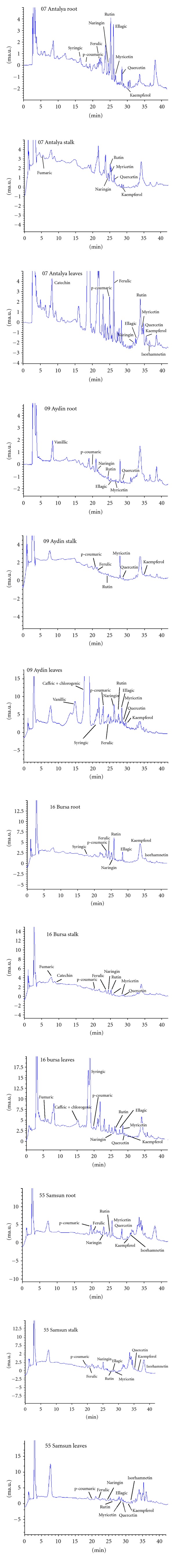
HPLC chromatogram of Antalya, Aydın, Bursa and Samsun root, stalk and leaves sample.

**Figure 2:**
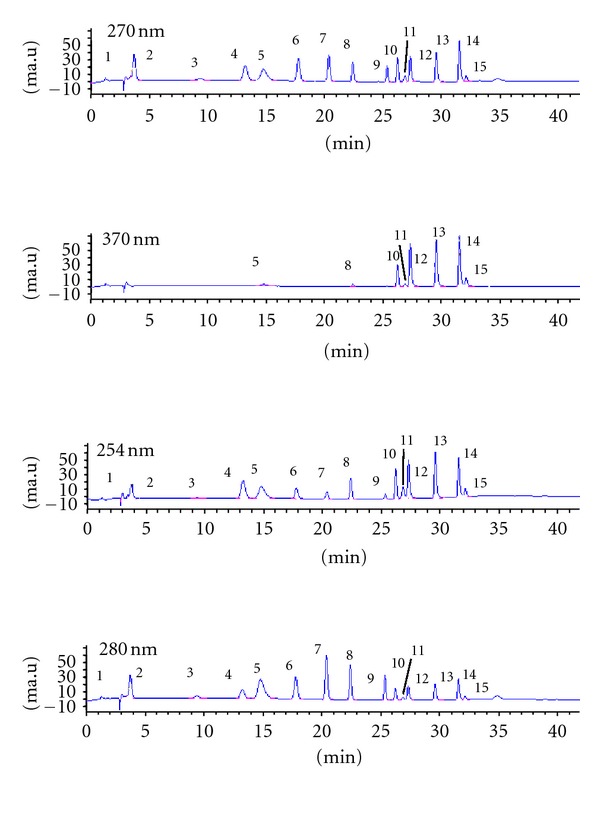
HPLC chromatogram of antioxidant standards. *1: fumaric acid; 2: gallic acid; 3: catechins; 4: vanillic acid; 5: caffeic + chlorogenic acid; 6: syringic acid; 7: p-coumaric acid; 8: ferulic; 9: naringin; 10: rutin; 11: ellagic; 12: myricetin; 13: quercetin; 14: kaempferol; 15: isorhamnetin.

Qualitative and quantitative analyses of caffeic acid, vanillic acid, naringin, syringic acid, and ferulic acid, ellagic acid, myricetin, kaempferol, isorhamnetin, catechin, chlorogenic acid, p-coumaric acid, rutin, fumaric and gallic acid component in nettle samples were done by HPLC. HPLC analysis was done by using graded elution program. The elution program could be summarized as follows, 0–11 min, 100% A, 30–40 min, 35% A, and 65% B, and 42 min 100% A. A and B solvents were used as elution solvent. Solvent A consists of 2% acetic acid, 10% methanol, and 88% pure water, and B consists of 2% acetic acid, 90% methanol, and 8% pure water. The flow rate was 1 mL/min, temperature was 40°C, and the injection volume was 20 *μ*L. On the other hand, according to maximum absorbance of standards, analysis was done at 254, 270, 280, and 370 nm wavelength. Some chromatogram samples are given at Figure 1. The standards chromatogram is given at Figure 2.

In HPLC analysis of nettle samples, the methanolic extracts were used. Samples and standards were filtered from 0,45 *μ*m Agilent micro filter, then were put into vials, and finally they were given to HPLC. 

## 3. Results and Discussion

### 3.1. Moisture Contents

Moisture content analysis of root, stalk, leaves, and total of nettle was done. This moisture analysis was given in the table (Tables 1 and 2) as region. Analysis was duplicated. The results were given with standard deviations. The differences between samples were determined by using SPSS (v.17)/one-way-aNOVA/Duncan test (*P* < 0.001).

According to scientific studies, moisture analysis was done to a nettle tea bag which includes all parts of nettle and as a result its moisture content was 6,3% [26]. In another study about nettle leaves which was collected from Macedonian, moisture content was 8,3% [1]. In another research about nettles (root, stalk and leaves), moisture contents of nettles root, stalk and leaves were 80,01%, 88,88%, and 78,67%, respectively [27].

In our study, average moisture content of nettles in total samples was 80,94%, root samples 81.87%, stalk samples 83,11%, and leaves samples 77,75%. The highest moisture content was 09 and the lowest one was 16 in total. In root the highest moisture content was 09Y and the lowest one was 53. In stalk the highest moisture content was 09Y and the lowest one was 16. In leaves the highest moisture content was 32 and the lowest one was 07F. On the other hand, the moisture content was different among the regions. As a general comment moisture content could be ranged like stalk > root > leaves.

Nettle samples, which were used in this study, are fresh samples that could be a reason of the high moisture content results.

### 3.2. Total Phenolic Content Analysis of Nettle

According to the analysis result (Tables 1 and 2), total phenolic content of nettle was indicated with FC method, which is a wide used method. Nettles (total, root, stalk, and leaves) were analyzed and this analysis result was given at 760 nm wavelengths. SPSS (v.17) statistical program, one-way-aNOVA/Duncan test (*P* < 0,001) was used to indicate the difference among samples. As a general comparison among samples, total phenolic content of nettle could be ranged as leaves > root > stalk.

According to a scientific research, total phenolic content analyses of nettle parts (root, stalk, and leaves) were done by using FC method. The results were given as mg Gallic Acid Equivalent (GAE)/g Dry Matter (DM). In this paper, the results were given as root 7,82, stalk 9,91, and leaves 7,62 mg GAE/g DM [27]. In comparison, our nettle samples total phenolic content is higher than this research results. In a research about phenolic analysis of nettle as cultivated and wild, total phenolic contents of nettles were given for stalk of nettle 28,6% in cultivated and 24,4% in wild samples, while 71,5% and 76,5% for leaves of nettle, respectively [28].

In another research, total phenolic content of nettle tea bag which includes all parts of nettle (root, stalk, and leaves) was 2,5 mg GAE/g DM [26].

### 3.3. Total Antioxidant Activity Analysis

Antioxidant activity analysis of nettle parts (root, stalk, and leaves) was done by using DPPH antioxidant activity method. The analysis result was given at 515 nm wavelengths. The results are given in Tables 1 and 2. SPSS (v.17) statistical program, one-way-aNOVA/Duncan test (*P* < 0,001) was used to indicate the difference among samples.

In a research related to nettle parts (root, stalk, and leaves), antioxidant activities were given as root 9,86, stalk 37,56, and leaves 76,06 mg GAE/g DM [27]. On the other hand, in another study about herbal tea, a nettle tea bag (include root, stalk, and leaves of nettle) total antioxidant activity was 2,5 mg GAE/g DM [26]. The process of nettle to prepare tea could be the reason of the lower antioxidant activity instead of fresh nettle parts. In a general perspective, antioxidant activity could be ranged as root > stalk > leaves. The 41 sample has the highest total antioxidant activity, and the 20 sample has the lowest one. In root sample, the highest one was 16 and the lowest one was 20. In stalk sample, the highest one was 41 and the lowest one was 09Y. In leaves sample, the highest one was 09 and the lowest one was 53.

The total antioxidant activity of nettle parts (root, stalk and leaves) was analyzed by using DPPH method. While the results were compared with literatures total antioxidant activity of fresh nettle was higher than the others (nettle tea, dry nettle leaves). According to the DPPH analysis results statistical discrepancy were observed between Mediterranean, Aegean, Black Sea, Marmara Region, root, stalk and leaves. On the other hand, there was no statistical discrepancy between Mediterranean leaves sample, Black Sea root, and stalk sample.

### 3.4. HPLC Analyses of Phenolic Component of Nettle

The total phenolic content and antioxidant activity of nettle samples which were collected from different regions and cities of Turkey were diverse between regions, cities, root, stalk and leaves. Nettles (total, root, stalk, and leaves) were analyzed and the results are given in Tables 3, 4, and 5. 16 antioxidant standards were used for identification of phenolic component of nettle sample in this research. Samples methanolic extracts were analyzed with these standards by HPLC-DAD. SPSS (v.17) statistical program, one-way-aNOVA/Duncan test (*P* < 0,001) was used to indicate the difference among samples.

According to the result, total phenolic components of nettle samples were considerably high by comparison of other researches. The analysis results have statistical discrepancy between regions, cities, and parts of nettles (root, stalk, and leaves). Nettle parts (root, stalk, and leaves) phenolic content analysis was done qualitative and quantitative by using HPLC-DAD. 

According to the results, there were not any gallic acid, syringic, fumaric, vanillic, isorhamnetin, catechin, caffeic, and chlorogenic acid in the root samples from Mediterranean region, but there were myricetin, rutin, ellagic acid, ferulic, and naringin. These standards have statistical discrepancy. There were not any gallic acid, vanillic, and catechin in stalk samples, but there were myricetin, isorhamnetin, ferulic and naringin. These standards have statistical discrepancy. There were not gallic acid, fumaric, and catechin in leaves samples, but there were myricetin, quercetin, rutin, ellagic, caffeic, and chlorogenic acid. These standards have statistical discrepancy.

There were not gallic acid, fumaric, vanillic, catechin, caffeic, and chlorogenic acid in root samples from Aegean region; other standards were found in these samples. There were not gallic acid, fumaric, catechin, caffeic, and chlorogenic acid, but there were syringic, quercetin, kaempferol, and isorhamnetin in stalk samples. There were not gallic acid, fumaric, and catechin, but there were quercetin and p-coumaric acid in leaves samples.

There were not gallic acid, syringic, fumaric, vanillic, catechin, caffeic, and chlorogenic acid, but other standards were found in root samples from Black Sea region. There were not gallic acid, syringic, fumaric, vanillic, catechin, caffeic, and chlorogenic acid in stalk samples, but there were kaempferol, isorhamnetin, and naringin. There were not gallic acid, fumaric, vanillic, catechin, caffeic, and chlorogenic acid in leaves samples, but there was statistical discrepancy in quercetin and fumaric acid.

There were not gallic acid, vanillic, catechin, caffeic, and chlorogenic acid in Marmara region root samples, but other standards were found. There were not gallic acid, kaempferol, vanillic, catechin, ellagic, isorhamnetin, caffeic, and chlorogenic acid in stalk samples, but other standards were found. There were not gallic acid, vanillic, isorhamnetin, catechin, caffeic, and chlorogenic acid in leaves samples, but other standards were found.

By comparison of root samples, p-coumaric, kaempferol, and quercetin have not statistical discrepancy. On the other hand, there were no gallic acid, fumaric, vanillic, catechin, caffeic, and chlorogenic acid. By comparison of stalk samples, syringic, myricetin, quercetin, kaempferol, and rutin have not statistical discrepancy. On the other hand, there were not gallic acid, vanillic acid, catechin, caffeic, and chlorogenic acid. By comparison of leaves samples, p-coumaric, isorhamnetin, and quercetin have not statistical discrepancy. On the other hand, there were not gallic acid, fumaric acid, and catechin.

In a research about cultivated and wild nettle samples phenolic profile and HPLC analysis, caffeic acid derivative, chlorogenic acid, 2-O-caffeoylmalic acid, rutin, quercetin 3-O-glucoside, kaempferol 3-O-rutinoside, and isorhamnetin 3-O-rutinoside were found in cultivated leaves samples. Caffeic acid derivative, p-coumaric acid, caffeoylquinic acid, chlorogenic acid, rutin, quercetin 3-O-glucoside, kaempferol 3-O-rutinoside, and isorhamnetin 3-O-rutinoside, peonidin 3-O-rutinoside, peonidin 3-O-(6′′-O-p-coumaroylglucoside), and rosinidin 3-O-rutinoside were found in cultivated stalk samples. Caffeic acid derivative, p-coumaric acid, chlorogenic acid, 2-O-caffeoylmalic acid, rutin, quercetin 3-O-glucoside, kaempferol 3-O-rutinoside, isorhamnetin 3-O-rutinoside were found in wild leaves samples. Finally, caffeic acid derivative, p-coumaric acid, caffeoylquinic acid, chlorogenic acid, rutin, quercetin 3-O-glucoside, kaempferol 3-O-rutinoside, isorhamnetin 3-O-rutinoside, peonidin 3-O-rutinoside, rosinidin 3-O-rutinoside were found in wild stalk samples [28]. According to our results, there were not any gallic acid, vanillic, and catechin in stalk samples, but there were myricetin, isorhamnetin, ferulic, and naringin in the stalk samples from Mediterranean region. There were not gallic acid, fumaric, catechin, caffeic, and chlorogenic acid, but there were syringic, quercetin, kaempferol, and isorhamnetin in stalk samples from Agean region. There were not gallic acid, syringic, fumaric, vanillic, catechin, caffeic, and chlorogenic acid in stalk samples from Black Sea region, but there were kaempferol, isorhamnetin, and naringin. There were not gallic acid, kaempferol, vanillic, catechin, ellagic, isorhamnetin, caffeic, and chlorogenic acid in stalk samples from Marmara region, but other standards were found. There were not gallic acid, fumaric, and catechin, but there were myricetin, quercetin, rutin, ellagic, caffeic, and chlorogenic acid in leaves samples from Mediterranean region. There were not gallic acid, fumaric, and catechin, but there were quercetin, and p-coumaric acid in leaves samples from Agean region. There were not gallic acid, fumaric, vanillic, catechin, caffeic, and chlorogenic acid in leaves samples from Black Sea region, but there was statistical discrepancy in quercetin, and fumaric acid. There were not gallic acid, vanillic, isorhamnetin, catechin, caffeic, and chlorogenic acid in leaves samples from Marmara region, but other standards were found.

In another research, some kinds of plants HPLC-MS analysis were done. One of these plants was nettle, and according to the results nettles included 18 ppm quercetin, 13 ppm myricetin, and 7,7 ppm kaempferol [29]. In comparison to our study, root, stalk, and leaves parts of nettle have lower phenolics components than those in this study, as seen in Tables 1 and 2.

## 4. Conclusions

Nettle is a plant easy to grow. Nettle is rich of chemical component and composition. It is as widely used from cosmetics to food. It is widely used plant in complementary and alternative treatment method (CTM and ATM) as cancer, and so forth. It is thought that nettle has these positive properties as its phenolic contents. Also, these plant parts (root, stalk, and leaves) have different phenolic composition and contents. The nettle part content differences indicate that different parts could be used for different cancers in ATM. Because of that reason, the phenolic compounds and contents of nettle parts were tried to identify. By this way, different kinds of phenolic components of nettles part (root, stalk, and leaves) indicate different usage area to the plant parts. Phenolic content analyses were done by using nettle parts (root, stalk and leaves) and the nettles were collected from different regions of Turkey (Aegean, Black Sea, Mediterranean, Marmara), according to these properties of nettle.

Nettle prefers nutrient riches and lighted places, hot and mild climate. Therefore, the higher total phenolic contents and antioxidant activities of nettles and roots of nettles were found in Marmara and Black Sea region in this research, while the higher moisture contents of nettles, roots, and stalks of nettles were found in Aegean and Mediterranean region. The higher moisture contents of leaves were found in Aegean and Black Sea region. The higher total phenolic contents of stalk samples was found in Black Sea and Mediterranean region, while the higher total antioxidant activities of stalk samples were found in Marmara and Black Sea region. The higher total phenolic contents of leaves samples were found in Marmara and Aegean region, while the higher total antioxidant activity of leaves samples were found in Marmara and Mediterranean region. According to analysis results, nettles phenolic compounds and contents were different between regions and parts of nettles. The difference between parts of nettle shows that all the parts of nettle could have different usages. In comparison of nettle tea and dried nettle from other researches, fresh samples phenolic contents were higher than others. For that reason, fresh nettle consumption could be healthier.

## Figures and Tables

**Table 1:** Moisture content, total phenolic content, and total antioxidant activity of total and root of fresh nettle.

	Moisture %	FC (mg GAE/g DM)	DPPH (mg GAE/g DM)
*Sample*			
M01	84,61 ± 0,05^b^	157,27 ± 5,33^b^	147,20 ± 2,09^a^
M07	74,93 ± 0,04^c^	307,10 ± 1,12^a^	241,63 ± 2,72^a^
M07F	74,03 ± 0,02^c^	344,12 ± 9,19^a^	247,16 ± 1,14^a^
M32	89,74 ± 0,02^a^	123,62 ± 1,68^b^	98,70 ± 1,35^b^
A09Y	89,80 ± 0,11^a^	243,68 ± 3,35^c^	51,44 ± 2,74^b^
A09	88,84 ± 0,18^a^	113,69 ± 2,87^d^	145,83 ± 0,39^a^
A20	88,88 ± 0,21^a^	730,65 ± 5,85^d^	52,78 ± 0,48^b^
A35	84,09 ± 0,06^b^	126,95 ± 1,07^a^	141,54 ± 1,14^a^
A45A	80,74 ± 0,00^c^	277,25 ± 3,65^c^	114,04 ± 1,30^a^
A45S	86,96 ± 0,21^a,b^	123,88 ± 0,98^d^	53,27 ± 0,31^b^
A45T	85,56 ± 0,15^b^	383,93 ± 3,96^b^	72,73 ± 0,35^b^
A48	89,08 ± 0,15^a^	66,97 ± 0,89^d^	114,11 ± 0,47^a^
B52	76,29 ± 0,11^b^	440,49 ± 16,06^b^	106,69 ± 0,91^a^
B53	78,45 ± 0,04^a^	233,93 ± 5,16^b^	235,38 ± 2,19^a^
B55	69,66 ± 0,05^c^	723,93 ± 37,56^a^	152,26 ± 1,05^a^
B61	78,64 ± 0,03^a^	353,92 ± 11,34^b^	224,87 ± 1,92^a^
B74	78,10 ± 0,02^a,b^	297,15 ± 2,90^b^	234,18 ± 1,16^a^
MS16	62,55 ± 0,05^b^	410,53 ± 3,21^b^	260,92 ± 1,01^a^
MS41	76,43 ± 0,03^a^	443,96 ± 6,33^a^	249,96 ± 1,91^a^
*Root Sample*			
M01	87,89 ± 0,02^b^	70,47 ± 1,14^c^	150,85 ± 0,42^a^
M07	74,96 ± 0,08^a^	370,12 ± 0,93^a^	300,60 ± 1,83^a^
M07F	77,92 ± 0,01^a^	170,99 ± 4,39^b^	250,81 ± 0,95^a^
M32	89,79 ± 0,04^b^	20,44 ± 0,24^c^	130,01 ± 2,35^a^
A09Y	93,45 ± 0,05^a^	60,03 ± 0,34^a^	30,60 ± 1,16^b^
A09	91,31 ± 0,19^a^	20,12 ± 0,31^b^	50,60 ± 0,12^b^
A20	89,96 ± 0,10^a,b^	100,89 ± 0,18^b^	40,22 ± 0,20^b^
A35	86,36 ± 0,07^b,c^	90,86 ± 1,56^b^	170,42 ± 1,96^a^
A45A	84,35 ± 0,00^c^	40,49 ± 0,45^b^	90,74 ± 0,01^a^
A45S	89,46 ± 0,53^a,b^	90,94 ± 2,24^b^	40,31 ± 0,19^b^
A45T	86,39 ± 0,08^b,c^	90,35 ± 2,53^b^	60,97 ± 0,55^b^
A48	70,45 ± 0,26^a^	390,33 ± 9,60^b^	120,24 ± 0,51^a^
B52	71,38 ± 0,08^a^	790,21 ± 43,65^a^	140,58 ± 2,46^a^
B53	70,44 ± 0,01^a^	40,52 ± 0,13^a^	340,31 ± 1,31^a^
B55	73,56 ± 0,11^a^	1020,16 ± 69,40^a^	190,91 ± 2,35^a^
B61	78,09 ± 0,00^a^	270,62 ± 8,87^a^	350,72 ± 1,69^a^
B74	92,32 ± 0,01^a^	190,67 ± 0,36^a^	270,62 ± 0,67^a^
MS16	71,42 ± 0,03^b^	430,34 ± 6,20^b^	370,27 ± 0,11^a^
MS41	76,12 ± 0,00^a^	470,78 ± 0,46^a^	300,15 ± 2,13^a^

*M is representing Mediterranean, A is Aegean, B is Black Sea, and MS is Marmara region. The statistical analysis was done within Region.

**Table 2:** Moisture content, total phenolic content, and total antioxidant activity of stalk and leaves of fresh nettle.

	Moisture %	FC (mg GAE/g DM)	DPPH (mg GAE/g DM)
*Stalk Sample*			
M01	85,29 ± 0,02^b^	20,24 ± 0,68^b,c^	90,06 ± 0,52^b^
M07	84,11 ± 0,01^b,c^	80,46 ± 2,46^b^	300,61 ± 2,40^a^
M07F	81,32 ± 0,04^c^	150,35 ± 2,16^a^	180,39 ± 1,72^a^
M32	92,81 ± 0,02^a^	370,58 ± 8,51^c^	230,39 ± 5,16^a^
A09Y	93,40 ± 0,20^a^	10,08 ± 0,06^e^	30,31 ± 0,19^b^
A09	91,17 ± 0,30^b,c^	60,71 ± 1,34^c^	50,77 ± 0,15^b^
A20	91,62 ± 0,03^a,b^	150,07 ± 2,31^b^	40,85 ± 0,48^b^
A35	87,85 ± 0,02^d^	100,42 ± 0,64^a^	140,15 ± 0,91^a^
A45A	84,66 ± 0,05^e^	70,49 ± 1,60^b,c^	130,82 ± 0,42^a^
A45S	90,71 ± 0,01^b,c^	40,66 ± 2,03^c,d^	40,29 ± 0,17^b^
A45T	89,15 ± 0,09^c,d^	60,25 ± 0,59^c,d^	70,19 ± 0,07^b^
A48	77,84 ± 0,12^c,d^	30,28 ± 0,96^d,e^	30,71 ± 0,68^b^
B52	79,41 ± 0,08^a^	160,72 ± 3,47^a^	90,41 ± 0,15^a^
B53	79,86 ± 0,13^a^	320,85 ± 4,53^a^	290,63 ± 3,65^a^
B55	80,36 ± 0,01^a^	270,62 ± 28,61^a^	90,35 ± 0,10^a^
B61	77,48 ± 0,05^a^	260,61 ± 6,84^a^	240,45 ± 3,04^a^
B74	89,41 ± 0,03^a^	480,30 ± 3,33^a^	300,93 ± 2,01^a^
MS16	43,78 ± 0,04^b^	120,71 ± 3,38^a^	240,86 ± 1,48^a^
MS41	78,91 ± 0,05^a^	100,90 ± 4,79^b^	320,99 ± 3,44^a^
*Leaves Sample*			
M01	86,63 ± 0,02^b^	320,88 ± 12,39^a^	110,39 ± 4,14^a^
M07	80,65 ± 0,11^c^	400,83 ± 0,26^a^	190,04 ± 1,15^a^
M07F	65,72 ± 0,02^c^	490,80 ± 14,67^a^	170,79 ± 0,07^a^
M32	62,84 ± 0,01^a^	330,20 ± 4,12^a^	70,54 ± 1,20^b^
A09Y	82,57 ± 0,10^a^	330,61 ± 0,38^d^	80,53 ± 6,89^a^
A09	84,04 ± 0,04^a^	190,01 ± 4,74^d,e^	320,38 ± 0,89^a^
A20	85,07 ± 0,48^a^	220,40 ± 2,22^d,e^	60,77 ± 0,75^a^
A35	78,07 ± 0,09^a,b^	1941,00 ± 15,06^a^	100,90 ± 0,53^a^
A45A	80,59 ± 0,12^b^	150,52 ± 1,41^c^	80,17 ± 0,49^a^
A45S	80,72 ± 0,01^a^	1000,61 ± 9,06^d,e^	100,56 ± 0,34^a^
A45T	81,13 ± 0,25^a^	670,40 ± 7,78^b^	170,28 ± 3,81^a^
A48	73,22 ± 0,00^a^	290,50 ± 0,45^e^	70,96 ± 0,06^a^
B52	84,56 ± 0,14^a^	370,54 ± 1,06^b^	80,02 ± 0,11^a^
B53	58,68 ± 0,11^a^	340,43 ± 10,82^b^	60,62 ± 1,60^a^
B55	81,99 ± 0,00^b^	881,00 ± 14,67^a^	160,43 ± 0,71^a^
B61	78,74 ± 0,06^a^	530,52 ± 18,32^b^	70,29 ± 1,02^a^
B74	85,51 ± 0,04^a^	220,49 ± 5,01^b^	110,71 ± 0,79^a^
MS16	72,44 ± 0,09^b^	680,55 ± 0,04^b^	160,14 ± 1,44^a^
MS41	74,25 ± 0,04^a^	760,20 ± 13,75^a^	110,85 ± 0,15^a^

*M is representing Mediterranean, A is Aegean, B is Black Sea, and MS is Marmara region. The statistical analysis was done within region.

**Table:** (a)

Sample	Gallic	Syringic	Myricetin	Quercetin	Kaempferol	Fumaric	Vanillic
M01	—	—	1,45 ± 0,13^a,b^	0,74 ± 0,02^a^	0,49 ± 0,11^a^	—	—
M07	—	—	0,64 ± 0,014^b^	0,84 ± 0,10^a^	0,47 ± 0,19^a^	—	—
M07F	—	—	2,18 ± 0,079^a,b^	0,73 ± 0,01^a^	0,52 ± 0,52^a^	—	—
M32	—	—	4,90 ± 1,83^a^	0,75 ± 0,01^a^	0,46 ± 0,01^a^	—	—
A09Y	—	0,00 ± 0,00^b^	0,70 ± 0,08^b^	0,38 ± 0,54^b^	0,44 ± 0,01^a^	—	—
A09	—	0,00 ± 0,00^b^	1,13 ± 0,34^b^	0,78 ± 0,08^a,b^	0,25 ± 0,35^a^	—	—
A20	—	4,31 ± 2,74^a^	21,93 ± 3,69^a^	0,77 ± 0,07^a,b^	0,46 ± 0,01^a^	—	—
A35	—	0,31 ± 0,43^b^	0,89 ± 0,08^b^	0,76 ± 0,02^a,b^	0,24 ± 0,34^a^	—	—
A45A	—	0,00 ± 0,00^b^	0,64 ± 0,01^b^	0,78 ± 0,09^a,b^	0,45 ± 0,03^a^	—	—
A45S	—	0,00 ± 0,00^b^	0,75 ± 0,02^b^	0,73 ± 0,01^a,b^	0,22 ± 0,32^a^	—	—
A45T	—	0,00 ± 0,00^b^	4,36 ± 2,76^b^	1,15 ± 0,33^a^	0,51 ± 0,09^a^	—	—
A48	—	0,00 ± 0,00^b^	0,34 ± 0,48^b^	0,71 ± 0,01^a,b^	0,22 ± 0,31^a^	—	—
B52	—	—	0,64 ± 0,02^a^	0,73 ± 0,00^a^	0,23 ± 0,32^a^	—	—
B53	—	—	0,49 ± 0,69^a^	0,76 ± 0,01^a^	0,24 ± 0,33^a^	—	—
B55	—	—	0,71 ± 0,06^a^	0,84 ± 0,14^a^	0,66 ± 0,09^a^	—	—
B61	—	—	0,69 ± 0,01^a^	0,76 ± 0,05^a^	0,51 ± 0,08^a^	—	—
B74	—	—	0,69 ± 0,02^a^	0,74 ± 0,01^a^	0,46 ± 0,02^a^	—	—
MS16	—	2,99 ± 0,83^a^	0,00 ± 0,00^b^	0,38 ± 0,55^a^	0,24 ± 0,33^a^	6,28 ± 8,88^a^	—
MS41	—	0,00 ± 0,00^a^	0,66 ± 0,01^a^	0,75 ± 0,01^a^	0,54 ± 0,09^a^	0,00 ± 0,00^a^	—

*M is representing Mediterranean, A is Aegean, B is Black Sea, and MS is Marmara Region. The statistical analyses were done within region.

**Table:** (b)

Sample	Rutin	Ellagic	Isorhamnetin	Catechin	Caffeic + chlorogenic	p-coumaric	Ferulic	Naringin
M01	6,38 ± 4,10^b^	6,50 ± 1,62^b^	—	—	—	1,86 ± 0,32^a^	1,15 ± 1,14^b^	2,66 ± 0,23^b^
M07	3,93 ± 0,80^b^	1,86 ± 1,86^b^	—	—	—	3,37 ± 2,01^a^	10,18 ± 1,01^a^	37,97 ± 5,67^a^
M07F	59,60 ± 20,79^a^	2,03 ± 2,03^b^	—	—	—	5,29 ± 2,38^a^	27,43 ± 10,52^a^	5,61 ± 1,61^b^
M32	0,00 ± 0,00^b^	26,81 ± 0,18^a^	—	—	—	0,00 ± 0,00^a^	0,39 ± 0,39^b^	0,00 ± 0,00^b^
A09Y	0,00 ± 0,00^b^	0,00 ± 0,00^b^	0,00 ± 0,00^b^	—	—	1,94 ± 0,33^b^	2,57 ± 0,41^b^	0,00 ± 0,00^d^
A09	0,92 ± 0,31^a,b^	3,51 ± 0,50^a,b^	3,42 ± 4,83^b^	—	—	3,16 ± 1,19^a,b^	0,00 ± 0,00^b^	0,49 ± 0,69^c,d^
A20	0,90 ± 0,27^a,b^	3,95 ± 5,59^a,b^	0,00 ± 0,00^b^	—	—	8,42 ± 5,60^a^	29,34 ± 3,06^a^	0,46 ± 0,06^a^
A35	0,73 ± 1,03^a,b^	0,00 ± 0,00^b^	84,85 ± 4,04^a^	—	—	3,83 ± 0,33^a,b^	0,00 ± 0,00^b^	2,52 ± 1,45^b,c,d^
A45A	0,49 ± 0,69^a,b^	0,00 ± 0,00^b^	0,00 ± 0,00^b^	—	—	5,06 ± 0,75^a,b^	0,00 ± 0,00^b^	3,10 ± 0,21^b,c^
A45S	0,00 ± 0,00^b^	0,00 ± 0,00^b^	27,63 ± 13,48^b^	—	—	2,53 ± 0,25^a,b^	0,00 ± 0,00^b^	0,00 ± 0,00^d^
A45T	0,98 ± 0,58^a,b^	12,92 ± 13,09^a^	0,00 ± 0,00^b^	—	—	3,00 ± 4,24^a,b^	0,99 ± 1,41^b^	0,87 ± 1,23^c,d^
A48	1,53 ± 0,52^a^	0,00 ± 0,00^b^	23,70 ± 33,52^b^	—	—	2,49 ± 0,69^a,b^	1,21 ± 1,71^b^	5,04 ± 2,45^a,b^
B52	1,71 ± 0,35^a^	10,74 ± 1,81^a^	17,49 ± 17,24^a^	—	—	3,71 ± 1,64^a^	4,26 ± 2,87^a^	9,84 ± 12,01^a^
B53	1,39 ± 0,64^a^	3,94 ± 0,05^a,b^	5,65 ± 8,00^a^	—	—	5,02 ± 0,83^a^	3,74 ± 4,80^a^	19,72 ± 11,27^a^
B55	4,20 ± 1,25^a^	3,59 ± 5,09^a,b^	15,29 ± 11,32^a^	—	—	8,34 ± 0,15^a^	11,27 ± 0,52^a^	20,49 ± 0,93^a^
B61	6,43 ± 5,60^a^	1,39 ± 1,97^b^	1,37 ± 1,93^a^	—	—	4,16 ± 1,77^a^	3,83 ± 1,05^a^	14,50 ± 4,68^a^
B74	4,53 ± 4,48^a^	5,39 ± 1,92^a,b^	0,37 ± 0,52^a^	—	—	6,99 ± 6,09^a^	7,06 ± 5,01^a^	10,15 ± 6,11^a^
MS16	2,43 ± 2,37^a^	2,01 ± 2,83^a^	5,47 ± 7,74^a^	—	—	3,23 ± 0,52^a^	4,23 ± 1,25^a^	13,56 ± 5,64^a^
MS41	2,55 ± 3,07^a^	2,03 ± 2,87^a^	10,91 ± 15,43^a^	—	—	4,76 ± 1,77^a^	6,05 ± 2,46^a^	17,31 ± 1,79^a^

*M is representing Mediterranean, A is Aegean, B is Black Sea, and MS is Marmara Region. The statistical analyses were done within region.

**Table:** (a)

Sample	Gallic	Syringic	Myricetin	Quercetin	Kaempferol	Fumaric	Vanillic
M01	—	0,00 ± 0,00^a^	1,15 ± 0,18^a,b^	0,72 ± 0,01^a^	0,24 ± 0,24^a^	6,18 ± 6,18^a^	—
M07	—	0,00 ± 0,00^a^	0,75 ± 0,08^b^	0,37 ± 0,37^a^	0,38 ± 0,38^a^	11,66 ± 0,99^a^	—
M07F	—	2,63 ± 1,94^a^	1,57 ± 0,21^a^	1,02 ± 0,05^a^	0,24 ± 0,24^a^	9,17 ± 1,32^a^	—
M32	—	0,00 ± 0,00^a^	0,66 ± 0,01^b^	0,76 ± 0,05^a^	0,26 ± 0,26^a^	2,09 ± 2,96^a^	—
A09Y	—	0,00 ± 0,00^a^	0,50 ± 0,72^b^	0,36 ± 0,51^a^	0,48 ± 0,05^a^	—	0,00 ± 0,00^b^
A09	—	0,00 ± 0,00^a^	0,76 ± 0,16^b^	0,75 ± 0,01^a^	0,44 ± 0,01^a^	—	0,00 ± 0,00^b^
A20	—	1,20 ± 1,69^a^	8,96 ± 11,65^a^	0,77 ± 0,05^a^	0,51 ± 0,05^a^	—	0,00 ± 0,00^b^
A35	—	2,49 ± 1,22^a^	1,26 ± 0,01^b^	0,78 ± 0,07^a^	0,45 ± 0,01^a^	—	39,46 ± 18,00^a^
A45A	—	0,00 ± 0,00^a^	4,61 ± 5,61^b^	0,77 ± 0,05^a^	0,45 ± 0,01^a^	—	0,00 ± 0,00^b^
A45S	—	49,28 ± 69,69^a^	1,85 ± 1,10^b^	4,54 ± 5,33^a^	0,31 ± 0,44^a^	—	0,00 ± 0,00^b^
A45T	—	0,00 ± 0,00^a^	3,70 ± 2,59^b^	0,79 ± 0,03^a^	0,25 ± 0,35^a^	—	0,00 ± 0,00^b^
A48	—	0,00 ± 0,00^a^	3,02 ± 2,14^b^	0,73 ± 0,01^a^	0,49 ± 0,04^a^	—	0,00 ± 0,00^b^
B52	—	—	0,78 ± 0,14^a^	0,74 ± 0,03^a^	0,63 ± 0,26^b^	—	—
B53	—	—	0,85 ± 0,22^a^	0,77 ± 0,02^a^	0,22 ± 0,32^b^	—	—
B55	—	—	0,76 ± 0,10^a^	0,73 ± 0,01^a^	0,52 ± 0,12^b^	—	—
B61	—	—	0,93 ± 0,40^a^	0,73 ± 0,03^a^	1,21 ± 0,20^a^	—	—
B74	—	—	0,67 ± 0,03^a^	0,74 ± 0,01^a^	0,47 ± 0,39^b^	—	—
MS16	—	1,12 ± 1,32^a^	0,32 ± 0,46^a^	0,73 ± 0,01^a^	—	8,18 ± 0,85^a^	—
MS41	—	0,00 ± 0,00^a^	0,71 ± 0,07^a^	0,32 ± 0,01^b^	—	0,00 ± 0,00^b^	—

*M is representing Mediterranean, A is Aegean, B is Black Sea, and MS is Marmara Region. The statistical analyses were done within region.

**Table:** (b)

Sample	Rutin	Ellagic	Isorhamnetin	Catechin	Caffeic + chlorogenic	p-coumaric	Ferulic	Naringin
M01	29,62 ± 26,66^a^	2,93 ± 2,92^a^	0,94 ± 0,94^a,b^	—	25,91 ± 23,73^a^	3,87 ± 2,55^a^	20,82 ± 1,37^a,b^	3,79 ± 0,77^b^
M07	7,72 ± 3,17^a^	1,96 ± 1,96^a^	0,00 ± 0,00^b^	—	0,00 ± 0,00^a^	0,00 ± 0,00^a^	0,00 ± 0,00^b^	8,81 ± 0,25^a^
M07F	6,41 ± 1,66^a^	3,82 ± 0,19^a^	3,14 ± 0,78^a^	—	17,18 ± 1,75^a^	8,07 ± 1,59^a^	44,50 ± 22,24^a^	6,05 ± 2,29^a,b^
M32	33,65 ± 33,65^a^	0,00 ± 0,00^a^	0,00 ± 0,00^b^	—	0,00 ± 0,00^a^	0,00 ± 0,00^a^	0,00 ± 0,00^b^	0,00 ± 0,00^c^
A09Y	0,00 ± 0,00^b^	0,00 ± 0,00^b^	0,00 ± 0,00^a^	—	—	1,64 ± 0,57^b^	4,44 ± 0,58^b^	0,00 ± 0,00^c^
A09	1,20 ± 0,24^b^	1,54 ± 2,17^b^	0,00 ± 0,00^a^	—	—	2,41 ± 0,21^b^	0,03 ± 0,05^b^	0,21 ± 0,30^c^
A20	1,61 ± 0,15^b^	1,90 ± 2,69^b^	5,81 ± 8,22^a^	—	—	0,00 ± 0,00^b^	0,12 ± 0,16^b^	0,00 ± 0,00^c^
A35	0,00 ± 0,00^b^	3,62 ± 0,04^b^	46,49 ± 0,10^a^	—	—	22,04 ± 6,32^a^	0,00 ± 0,00^b^	12,08 ± 0,24^a^
A45A	0,35 ± 0,50^b^	0,00 ± 0,00^b^	0,00 ± 0,00^a^	—	—	2,70 ± 1,65^b^	0,00 ± 0,00^b^	1,27 ± 0,77^b,c^
A45S	3,34 ± 4,72^b^	4,96 ± 7,01^b^	19,39 ± 21,21^a^	—	—	7,18 ± 10,16^b^	11,20 ± 15,84^a,b^	3,95 ± 3,38^b^
A45T	19,31 ± 0,14^a^	29,39 ± 1,82^a^	0,00 ± 0,00^a^	—	—	1,50 ± 2,13^b^	24,16 ± 11,28^b^	1,62 ± 2,29^b,c^
A48	1,76 ± 2,48^b^	2,35 ± 3,33^b^	34,96 ± 49,45^a^	—	—	2,79 ± 0,52^a,b^	8,77 ± 0,78^a,b^	1,08 ± 0,63^b,c^
B52	3,07 ± 1,85^a^	6,07 ± 3,80^a^	0,51 ± 0,71^b^	—	—	6,45 ± 1,79^a^	5,53 ± 0,00^a^	13,04 ± 15,16^b^
B53	1,32 ± 0,39^a^	4,06 ± 2,08^a^	11,03 ± 2,51^a,b^	—	—	8,88 ± 1,47^a^	2,04 ± 2,88^a^	12,91 ± 0,01^b^
B55	0,79 ± 1,12^a^	5,38 ± 2,16^a^	16,91 ± 15,55^a,b^	—	—	3,29 ± 0,34^a^	1,66 ± 1,95^a^	16,42 ± 5,41^b^
B61	9,32 ± 8,65^a^	7,85 ± 2,38^a^	30,02 ± 7,46^a^	—	—	9,69 ± 3,75^a^	5,68 ± 1,37^a^	63,19 ± 8,68^a^
B74	9,76 ± 2,49^a^	5,68 ± 2,76^a^	0,00 ± 0,00^b^	—	—	3,08 ± 0,77^a^	5,84 ± 1,46^a^	32,00 ± 10,27^b^
MS16	2,03 ± 0,75^a^	—	—	—	—	1,48 ± 0,11^a^	0,86 ± 1,21^a^	7,19 ± 0,22^a^
MS41	4,38 ± 3,72^a^	—	—	—	—	3,78 ± 1,19^a^	0,00 ± 0,00^a^	22,48 ± 12,20^a^

*M is representing Mediterranean, A is Aegean, B is Black Sea and MS is Marmara Region. The statistical analyses were done within Region.

**Table:** (a)

Sample	Gallic	Syringic	Myricetin	Quercetin	Kaempferol	Fumaric	Vanillic
M01	—	0,00 ± 0,00^a^	1,15 ± 0,33^b^	0,96 ± 0,13^b^	0,61 ± 0,15^a^	—	26,15 ± 36,98^a^
M07	—	23,10 ± 32,67^a^	0,75 ± 1,^48a,b^	1,37 ± 0,03^a,b^	0,60 ± 0,01^a^	—	0,00 ± 0,00^a^
M07F	—	2,78 ± 3,93^a^	1,57 ± 0,55^a^	1,78 ± 0,17^a^	0,61 ± 0,17^a^	—	0,42 ± 0,59^a^
M32	—	0,00 ± 0,00^a^	0,65 ± 0,01^b^	1,23 ± 0,50^a,b^	0,49 ± 0,06^a^	—	0,00 ± 0,00^a^
A09Y	—	16,20 ± 2,07^b^	0,79 ± 0,22^b^	0,91 ± 0,11^a^	5,96 ± 2,05^a^	—	0,00 ± 0,00^b^
A09	—	341,68 ± 21,04^a^	2,08 ± 1,45^b^	1,19 ± 0,42^a^	1,68 ± 0,03^b^	—	40,68 ± 10,78^b^
A20	—	0,00 ± 0,00^b^	1,91 ± 0,73^b^	3,48 ± 2,30^a^	0,75 ± 0,13^b^	—	0,00 ± 0,00^b^
A35	—	0,00 ± 0,00^b^	1,67 ± 0,35^b^	1,16 ± 0,16^a^	0,68 ± 0,32^b^	—	295,21 ± 82,59^a^
A45A	—	0,00 ± 0,00^b^	1,20 ± 0,33^b^	1,38 ± 0,82^a^	0,93 ± 0,39^b^	—	0,00 ± 0,00^b^
A45S	—	0,00 ± 0,00^b^	1,03 ± 0,28^b^	1,38 ± 0,24^a^	2,15 ± 2,16^b^	—	20,85 ± 4,61^b^
A45T	—	0,00 ± 0,00^b^	6,39 ± 0,56^a^	2,95 ± 2,23^a^	0,59 ± 0,16^b^	—	0,00 ± 0,00^b^
A48	—	4,05 ± 5,73^b^	1,12 ± 0,63^b^	1,77 ± 0,12^a^	1,34 ± 1,30^b^	—	0,00 ± 0,00^b^
B52	—	7,80 ± 2,20^a^	0,82 ± 0,16^a^	1,30 ± 0,27^a,b^	0,97 ± 0,76^a^	—	—
B53	—	5,04 ± 7,13^a^	0,91 ± 0,36^a^	1,28 ± 0,15^a,b^	0,74 ± 0,18^a^	—	—
B55	—	0,00 ± 0,00^a^	1,26 ± 0,76^a^	1,37 ± 0,43^a^	1,07 ± 0,03^a^	—	—
B61	—	5,24 ± 7,42^a^	0,75 ± 0,13^a^	0,85 ± 0,06^a,b^	0,76 ± 0,47^a^	—	—
B74	—	7,49 ± 5,46^a^	1,26 ± 0,25^a^	0,79 ± 0,02^b^	1,33 ± 0,27^a^	—	—
MS16	—	48,99 ± 12,66^a^	0,88 ± 0,29^a^	0,96 ± 0,20^a^	0,85 ± 0,32^a^	10,93 ± 10,86^a^	—
MS41	—	46,92 ± 5,20^a^	2,18 ± 0,83^a^	0,84 ± 0,15^b^	1,69 ± 1,10^a^	0,00 ± 0,00^a^	—

*M is representing Mediterranean, A is Aegean, B is Black Sea and MS is Marmara Region. The statistical analyses were done within Region.

**Table:** (b)

Sample	Rutin	Ellagic	Isorhamnetin	Catechin	Caffeic + chlorogenic	p-coumaric	Ferulic	Naringin
M01	21,85 ± 23,91^c^	4,65 ± 6,57^a,b^	7,00 ± 9,90^a^	—	60,89 ± 1,87^a^	3,34 ± 0,01^b^	1,66 ± 1,02^a^	7,35 ± 2,78^a^
M07	96,67 ± 10,09^b^	9,62 ± 1,87^a,b^	12,42 ± 17,16^a^	—	0,00 ± 0,00^b^	1,87 ± 0,44^b^	11,61 ± 16,42^a^	9,41 ± 0,46^a^
M07F	191,07 ± 35,23^a^	15,76 ± 4,11^a^	8,34 ± 11,53^a^	—	80,38 ± 21,43^a^	9,25 ± 3,72^a^	7,14 ± 2,86^a^	12,79 ± 5,21^a^
M32	0,80 ± 0,26^c^	0,00 ± 0,00^b^	0,00 ± 0,00^a^	—	4,77 ± 6,75^b^	0,00 ± 0,00^b^	0,00 ± 0,00^a^	11,62 ± 0,31^a^
A09Y	2,74 ± 1,14^b^	8,18 ± 11,56^b^	11,37 ± 16,09^a,b^	—	0,00 ± 0,00^b^	3,42 ± 1,12^a^	8,69 ± 7,73^b^	8,73 ± 4,59^b^
A09	60,74 ± 26,33^a^	29,91 ± 25,44^a,b^	0,00 ± 0,00^b^	—	61,63 ± 18,81^a^	2,94 ± 0,19^a^	20,36 ± 1,76^a^	34,04 ± 13,58^a^
A20	0,97 ± 0,25^b^	5,73 ± 0,47^b^	7,71 ± 0,36^a,b^	—	26,13 ± 36,96^b^	1,31 ± 1,85^a^	0,23 ± 0,33^c^	0,00 ± 0,00^b^
A35	5,78 ± 1,33^b^	17,44 ± 1,97^a,b^	32,81 ± 14,81^a^	—	0,00 ± 0,00^b^	12,80 ± 18,11^a^	1,06 ± 1,49^c^	24,08 ± 2,37^a^
A45A	0,71 ± 0,49^b^	5,53 ± 1,84^b^	12,45 ± 5,09^a,b^	—	0,00 ± 0,00^b^	2,25 ± 0,22^a^	0,00 ± 0,00^c^	2,92 ± 0,61^b^
A45S	2,39 ± 1,06^b^	6,06 ± 1,48^b^	24,55 ± 21,45^a,b^	—	0,00 ± 0,00^b^	0,00 ± 0,00^a^	0,00 ± 0,00^c^	2,68 ± 0,16^b^
A45T	6,64 ± 3,18^b^	42,35 ± 5,88^a^	0,70 ± 0,99^b^	—	0,00 ± 0,00^b^	4,28 ± 1,42^a^	3,36 ± 1,59^b,c^	6,26 ± 5,57^b^
A48	0,84 ± 1,19^b^	11,39 ± 16,11^b^	0,00 ± 0,00^b^	—	0,00 ± 0,00^b^	4,87 ± 0,14^a^	0,52 ± 0,22^c^	4,77 ± 0,13^b^
B52	7,29 ± 4,32^a^	10,73 ± 10,47^a^	46,91 ± 58,43^a^	—	—	4,70 ± 1,68^a^	2,54 ± 0,05^a^	4,05 ± 1,03^a^
B53	4,01 ± 0,39^a,b^	4,63 ± 1,91^a^	12,56 ± 10,05^a^	—	—	4,71 ± 1,51^a^	2,72 ± 3,06^a^	2,85 ± 1,12^a^
B55	3,85 ± 0,71^a,b^	9,14 ± 3,29^a^	11,52 ± 11,00^a^	—	—	4,12 ± 2,82^a^	3,35 ± 3,77^a^	5,29 ± 2,63^a^
B61	0,62 ± 0,88^b^	7,68 ± 3,80^a^	0,00 ± 0,00^a^	—	—	4,68 ± 4,75^a^	5,64 ± 5,55^a^	17,15 ± 18,45^a^
B74	8,27 ± 1,75^a^	10,21 ± 6,05^a^	10,04 ± 14,20^a^	—	—	5,12 ± 0,55^a^	0,28 ± 0,39^a^	9,48 ± 4,41^a^
MS16	20,43 ± 4,91^a^	5,14 ± 1,28^a^	—	—	—	1,54 ± 0,37^a^	2,43 ± 3,43^a^	10,24 ± 0,57^a^
MS41	16,97 ± 2,85^a^	17,78 ± 1,71^a^	—	—	—	1,80 ± 2,54^a^	4,71 ± 2,42^a^	17,65 ± 1,75^a^

*M is representing Mediterranean, A is Aegean, B is Black Sea and MS is Marmara Region. The statistical analyses were done within Region.
